# A Review on Recent Advances in Aloperine Research: Pharmacological Activities and Underlying Biological Mechanisms

**DOI:** 10.3389/fphar.2020.538137

**Published:** 2020-10-29

**Authors:** Haifeng Zhou, Junyi Li, Fei Sun, Faxi Wang, Mingyue Li, Yalan Dong, Heng Fan, Desheng Hu

**Affiliations:** ^1^ Department of Integrated Traditional Chinese and Western Medicine, Union Hospital, Tongji Medical College, Huazhong University of Science and Technology, Wuhan, China; ^2^ The Center for Biomedical Research, Key Laboratory of Organ Transplantation, Ministry of Education, NHC Key Laboratory of Organ Transplantation, Chinese Academy of Medical Sciences, Tongji Hospital, Tongji Medical College, Huazhong University of Science and Technology, Wuhan, China

**Keywords:** *Sophora* genus phytomedicine, traditional herbal medicine, pharmacological effects, biological mechanisms, aloperine

## Abstract

Aloperine, a quinolizidine-type alkaloid, was first isolated from the seeds and leaves of herbal plant, *Sophora alopecuroides L*. Empirically, *Sophora alopecuroides L*. is appreciated for its anti-dysentry effect, a property that is commonly observed in other *Sophora Genus* phytomedicines. Following the rationale of reductionism, subsequent biochemical analyses attribute such anti-dysentry effect to the bactericidal activity of aloperine. From then on, the multiple roles of aloperine are gradually revealed. Accumulating evidence suggests that aloperine possesses multiple pharmacological activities and holds a promising potential in clinical conditions including skin hyper-sensitivity, tumor and inflammatory disorders etc.; however, the current knowledge on aloperine is interspersed and needs to be summarized. To facilitate further investigation, herein, we conclude the key pharmacological functions of aloperine, and most importantly, the underlying cellular and molecular mechanisms are clarified in detail to explain the functional mode of aloperine.

## Introduction

Many herbal plants have been proved to offer medical benefits and widely applied in clinical practice for thousands of years (Xu et al., 2013). Despite of the observed therapeutic effect, the miscellaneous components and obscure drug targets hinder their further utilization. Dosage and safety issues are of huge concerns; in addition, unknown pharmaceutical properties may exist. Along with the development of modern medical science, researchers are paying more attention to traditional herbal medicine, which helps to elucidate the underlying pharmacological function and accelerate the process of new drug discovery.

The genus of *Sophora* (family *Fabaceae*) contains approximately 70 species, most of which are distributed in tropical and temperate zones serving as pesticides and/or nectariferous plants (Aly et al., 2019). Apart from these, several *Sophora* species including *Sophora flavescens Ait* (Ku shen) and *Sophora subprostrata* (Sang zhi huai) etc. were used as traditional herbal in Eastern Asian countries (He et al., 2015; Aly et al., 2019). According to the theory of traditional Chinese medicine (TCM), these natural products display various activities in “clearing heat”, “dispelling dampness”, “relieving pain and swelling” and “sedation and detoxification”. From the viewpoints of modern medicine, “heat evil” in TCM is generally caused by exogenous virus or bacterial infection or endogenous functional over-activity, and “dampness evil” is often linked to inflammation-related responses or inflammatory exudation (Ren et al., 2017). Furthermore, the so-called “dampness and heat” in TCM is actually a very broad concept that is associated with many basic pathophysiological processes. Therefore, *Sophora* genus could provide valuable resources for screening of active ingredients.

**FIGURE 1:**
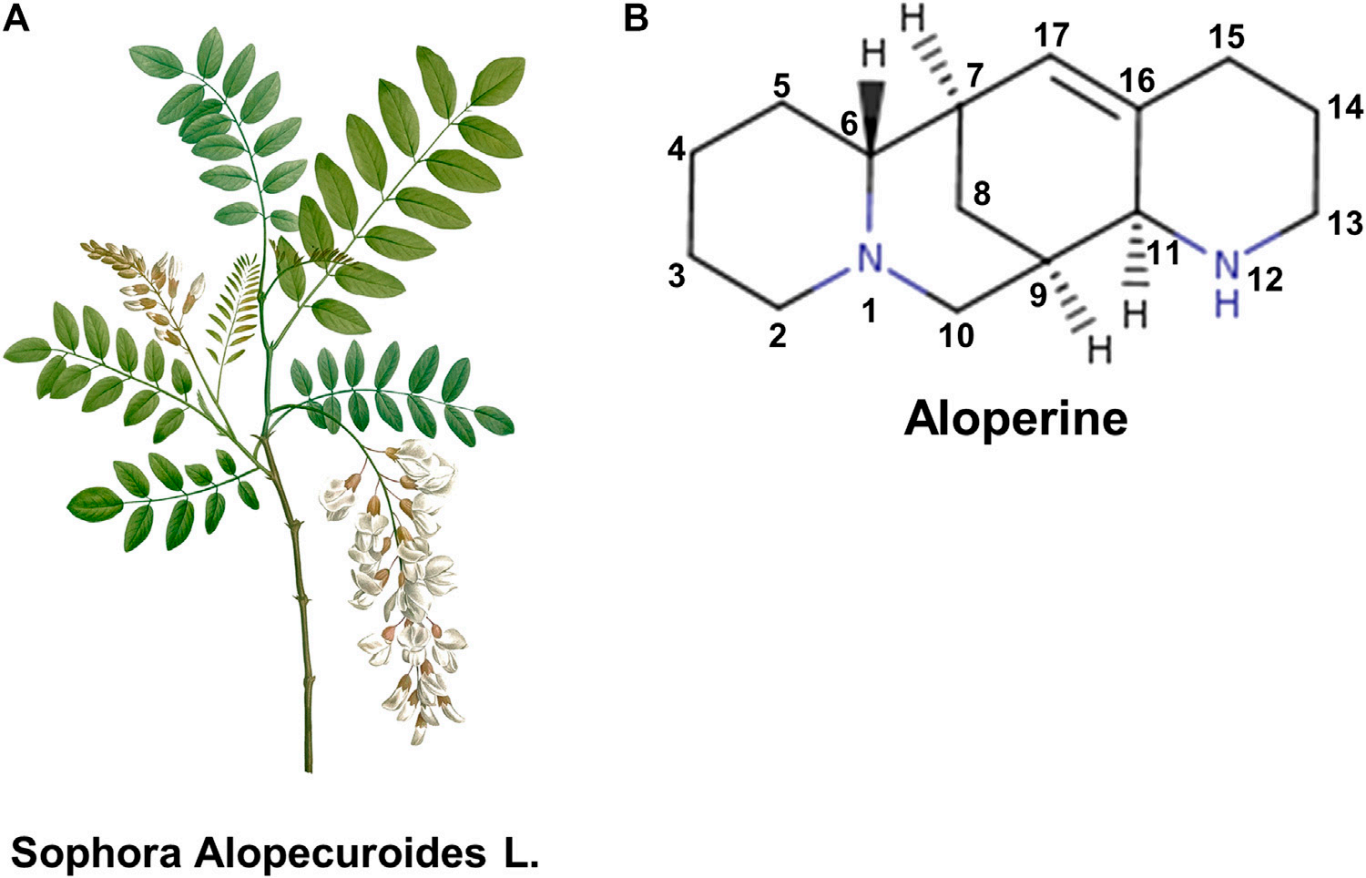
**(A)** Image of *sophora alopecuroids L.* **(B)** 2D chemical structure of aloperine (synthesized from piperidine-2-ethanol). C16-C17 double bond and N12 are important structural motifs that are commonly modified to improve the activities of aloperine.

Among the *Sophora* genus, *Sophora alopecuroides L.* (also known as Ku dou zi or Ku gan cao) mainly distributes in Western and Central Asia (Figure 1A), which is 1-m tall perennial herb with excellent capacity to withstand drought and alkali (Gao et al., 2011; Yugang and Hao, 2013). Based on the distinct feature of leaflets, *Sophora alopecuroides L.* can be divided into two major subspecies: one is *Sophora alopecuroides var. alopecuroides* (original variant) with appressed villous leaflets; another is *Sophora alopecuroides var. tomentosa* with densely patulous-tomentose ones. Strikingly, regardless of the subdivisions, almost all parts of the plant*,* including root, leaves and seeds, have been taken as medicinal herbs. Traditionally, *Sophora alopecuroides L.* was used effectively in the treatment of various clinical disorders, such as dysentery, eczema, recurrent dermatitis, furuncle, cancer and infectious diseases etc. (Gao et al., 2011; Wang et al., 2020). Chemical analysis revealed that *Sophora alopecuroides L.* contains many bioactive substances, including alkaloids, flavonoids, steroids and polysaccharides (Pu et al., 1987; Wu et al., 2018; Li Y. et al., 2020). Among these compounds, alkaloids are believed to make predominant contributions to the therapeutic effect of the herbal and thus have been extensively studied.

Aloperine was firstly discovered in 1935 as a natural alkaloid extracted from the seeds and leaves of *Sophora alopecuroides L*. Later on, it was also identified in other medical plants, such as *Sophorae flavescentis* and *Leptorhabdos parviflora Benth*. Purified aloperine is a white to yellowish crystalline powder with a molecular weight of 232.4, which can be stably stored at 4°C avoiding moisture and sunlight. It is soluble in organic solvents, such as ethanol and dimethyl formamide, but hard to dissolve in aqueous buffers. The identified absolute stereochemical structure and configuration of aloperine is of crucial importance for understanding the engaged biological processes and synthesis of aloperine based derivatives (Figure 1B) (Brosius and Overman, 1997; Brosius et al., 1997; Passarella et al., 2002; Yamauchi and Omi, 2005). Recently, a growing body of evidence from both basic science and clinical trials has demonstrated the pharmacological effects of aloperine. This review aims to summarize the current knowledge of aloperine mediated pharmaceutical activities with a focus on the mechanistic explanations, and hopefully, to also provide valuable insights for its clinical application.

## Antitumor Effect of Aloperine

In recent years, small natural compounds derived from herbal plants have drawn considerable attention due to their potential chemotherapeutic capacity and bio-safety (Cianciosi et al., 2018; Paier et al., 2018). Accumulative data demonstrated that aloperine itself and the modified derivatives display powerful antitumor effect on various cancer types both *in vivo* and *in vitro*. The ability of aloperine to target on multiple pathways makes it to be an extremely potent anticancer agent (Table 1).

**TABLE 1:** The effects and mechanisms of aloperine.

	Types	Model			Dose	Mechanisms	Target
Anti-tumor	Leukemia (Lin et al., 2011)	*In vitro*	—	HL-60, K562, U937	100 *μ*M	Induce apoptosis Induce autophagy Inhibit growth	(−) Bcl-2
	Prostate cancer (Ling et al., 2018)	*In vitro*	—	LNCaP, PC3, DU145	100 *μ*M, 200 *μ*M	Induce apoptosis induce cell cycle arrest	(+) p53/p21 (−) P-Akt, P-Erk (−) Bcl-2 (+) Bax (+)caspase3
		*In vivo*	(BALB/C node)	PC3	30 mg/kg		
	Hepatocellular carcinoma (Liu et al., 2019)	*In vivo*	(Zebrafish)	Huh7	50–150 *μ*M	Inhibit growth induce apoptosis induce G2/M cell cycle arrest	(−) PI3K/Akt release of cytochrome c (−) cdc25C, cdc2, cyclin B1
		*In vitro*	—	Hep3B, Huh7	200–500 *μ*M		
	Breast cancer (Tian et al., 2018)	*In vitro*	—	MCF7, MDA-MB-231	100 *μ*M, 200 *μ*M, 400 *μ*M	Inhibit proliferation induce apoptosis suppress migration and invasion	(+) caspase3,9 (−) Bcl-2 (+) Bax (−) MMP-2,9 (−) Ras/Raf1/Erk1/2
	Bladder cancer (Lv W. et al., 2020)	*In vitro*	-	T24	5 *μ*M, 10 *μ*M	Inhibit proliferation induce apoptosis. Suppress migration and invasion	(−) mTOR/p70S6K/4E-BP1
	Ovarian cancer	*In vitro*	—	A2780, SK-OV-3	50 *μ*g/mL, 100 *μ*g/mL, 200 *μ*g/mL, 500 *μ*g/mL	Induce apoptosis	(+) ROS
	Osteosarcoma (Chen et al., 2018)	*In vitro*	—	MG-63, U2OS	100 *μ*M, 200 *μ*M, 400 *μ*M	Induce apoptosis Antiproliferation Suppress migration and invasion	(−) PI3K/AKT (+) caspase3 (+) Bax (−) Bcl-2 (−) MMP-2,9
	Thyroid cancer (Lee et al., 2018)	*In vitro*	—	IHH-4, WRO, SW579, 8505c, KMH-2	200 *μ*M	Induce apoptosis inhibit growth	(−) PI3K/Akt
	Multiple myeloma (Wang et al., 2015)	*In vivo*	Xenograft mouse model, (C57/BL6) (KaLwRij	U266, 5T33 MM cells	20 mg/kg (p.o.)	Induce apoptosis inhibit growth	(−) cFLIP/caspase 8 (−) PTEN-Akt-caspase 9
		*In vitro*	—	Primary MM cells, U226, MM.1S, MM.1R, Dox6	80 *μ*M–10 mM		
	Colon cancer (Zhang et al., 2014)	*In vitro*	—	HCT116	250 *μ*M, 500 *μ*M, 1,000 *μ*M	Induce apoptosis antiproliferation induce G2/M cell cycle arrest	(−) JAK/Stat3 (−) PI3K/Akt (−) Bcl-2 (+) Bax (+) P21,P53 (−) cyclin B1, D1
	Non-small cell lung cancer (Zhang et al., 2019; Muhammad et al., 2020)	*In vitro*	—	H460, H1945, H157	10–40 *μ*M, 80–260 *μ*M	Activate cytotoxicity of NK and T cells by downregulating PD-L1 expression in cancer cells. Induce apoptosis	(+) PKCα-GSK3β (−) Bcl-2 (+) Bax
		*In vivo*	Lewis tumor xenograft mice model	LLCs	20 mg/kg, 50 mg/kg, 100 *μ*mg/kg (i.p.)		
Anti-microbial	Bacterial (Ho et al., 2016)	*P. gingivalis*			30 *μ*M	Inhibit adhesion Inhibit invasion	(−) firmA (+) microtubules arrangement (host)
	Virus (Dang et al., 2014; Dang et al., 2017; Zhang et al., 2018a; Zhang et al., 2018b; Lv X. et al., 2020)	HCV, HBV, HIVMARV, EBOV			10 *μ*M, 7 *μ*M	Inhibit invasion Block entry Disturb endocytosis	(−) Nucleoprotein (−) gp120 (−) Cathepsin B
Cardiovascular protection	Anti-atherosclerosis (Wang and Chen, 2006; Liu and Chen, 2014; Li W. et al., 2020)	*In vivo In vitro*	(SD rat)	HUVEC, U937	50 *μ*M, 100 *μ*M	Lower blood lipid level Inhibit monocytes to HUVECs	(−) IL-6, MCP-1, VCAM-1, E-selectin (−) Oxidative stress
	Anti-hypertension (Wu et al., 2017a; Brown et al., 2018; Yang et al., 2018; Chang et al., 2019; Manville et al., 2019)	*In vivo In vitro*	Monocrotaline (SD rat	Vascular muscle cells, HEK, PASMCs	25 mg/kg, 50 mg/kg, 100 mg/kg (i.g.) 300 *μ*M, 1 *μ*M, 500 *μ*M	Vasodilation inhibits vascular remodeling	Activate KCNQ5 (−) NF-κB, (−) cyclin E1 (+) p27^kip1^ (−)NOX-2/4 (−) RhoA/ROCK
	Myocardial protection (Mao et al., 2019)	*In vivo*	(SD rat)	—	200 mg/kg (i.g.)	Inhibit myocardial apoptosis Anti-arrhythmia	(+) PI3K/Akt
Anti-oxidation	Neuropathic pain (Xu et al., 2014)	*In vivo*	CCI (ICR)	—	25 mg/kg, 50 mg/kg, 80 mg/kg, 100 mg/kg (i.p.)	Reduce ROS	(−) NF-κB (+) SOD, GPx
	Alzheimer’s disease	*In vitro*	—	N2a/Swe.D9	50 *μ*M, 100 *μ*M	Reduce ROS and 4HNE	(+) GSH, GPx
	Brain injury (Ma et al., 2015; Song et al., 2018)	*In vivo In vitro*	SAH, OGD/RF (SD rat)	Primary hippocampal neuronal cells	75 mg/kg, 150 mg/kg (i.p.) 50 mg/L, 100 mg/L	Reduce ROS	(−) Nrf2-ARE
	Renal injury (Hu et al., 2016)	*In vivo In vitro*	Ischemia reperfusion (C57BL/6)	—	50 mg/kg (i.g.) 500 *μ*M	Reduce ROS	(+) SOD (+) Bcl-2/Bax
	Pulmonary fibrosis (Yin et al., 2018)	*In vivo*	Bleomycin (C57BL/6	—	40 mg/kg (i.g.)	Inhibit proliferation and differentiation of fibroblast	(−) PI3K/AKT/mTOR (−) TGF-β/Smad
Immuno-regulatory	Allergic airway inflammation (Wang et al., 2018)	*In vivo*	OVA (BALB/c	—	100 mg/kg, 200 mg/kg (i.g.)	Reducing goblet cell hyperplasia Inhibit inflammatory cells infiltration	(−) IL-4, IL-5 and IL-13
	Allergic contact dermatitis (Yuan et al., 2010; Yuan et al., 2011)	*In vivo*	DNFB (BALB/cNC/Nga)	—	1% (ad us.ext)	Inhibit inflammatory cells infiltration	(−) TNF-α, IL-1β and IL-6
	Colitis model (Fu et al., 2017)	*In vivo In vitro*	DSS (C57BL/6)	T Cell	40 mg/kg (i.g.), 250 *μ*M	Promote Treg	(−) PI3K/Akt/mTOR
	(Wang et al., 2018; Ye et al., 2020)	*In vitro*	LPS	RAW264.7	50 *μ*M, 100 *μ*M	Suppress inflammation	(−) iNOS, COX-2

Note: (+), promote; (−), inhibit. ROS, Reactive oxygen species; OGD/RP, oxygen–glucose deprivation and reperfusion; CCL, chronic constriction injury; SD rat, Sprague Dawley rat; OVA, Ovalbumin; DSS, dextran sodium sulfate; DNFB, 2,4-dinitrofluorobenzene; EBOV, Ebola virus; MARV, Marburg virus, HCV, Hepatitis C virus; HIV, Human immunodeficiency virus; p.o., oral; i.p., intraperitoneal; i.g., intragastric; ad us.ext., external use.

### Aloperine Impedes Tumor Survival

Research conducted by Jiang’s group tested the *in vitro* anticancer activities of six different kinds of quinolizidine alkaloids derived from *Sophora flavescensa*, including sophoridine, aloperine, sophocarpine, matrine, oxymatrine and cytisine. They found aloperine exerts the most potent cytotoxic activity on leukemia cell lines HL-60, U937 and K562, oesophageal cancer EC109 cells, lung cancer cells, glioma cells, ovarian cancer cells and the hepatocellular carcinoma HepG2 cell line (Wang et al., 2015; Liu et al., 2019; Xu et al., 2019; Muhammad et al., 2020; Qiu et al., 2020b). The mechanism inferred that occurrence of apoptosis (fragmentation of DNA, cleavage of PARP and decreased protein level of Bcl-2) and formation of acidic vacuole, a marker of autophagy. These results suggested that apoptotic and autophagic pathways are critically engaged (Lin et al., 2011).

Specifically, the antitumor activity of aloperine on prostate LNCaP, PC3 and DU145 cancer cell lines was investigated *in vitro* and *in vivo*. Aloperine inhibits PI3K/Akt and Ras/Erk signaling pathway, thus inducing the expression of pro-apoptotic gene caspase-3, decreasing the ratio of Bcl-2/Bax and upregulating the level of tumor suppressor p53 and p21 (Ling et al., 2018). Song’s group pointed out that aloperine significantly inhibits the viability of bladder cancer cells via suppressing hypoxia induced activation of mTOR/p70S6K/4E-BP1 pathway (Lv W. et al., 2020). Besides, Liu et al. illustrated that aloperine induces apoptosis and G2/M cell cycle arrest in hepatocellular carcinoma cell lines Hep3B and Huh7 *in vitro*, and suppresses tumor development in the zebrafish xenograft model *in vivo* (Liu et al., 2019). Similar mechanisms were confirmed on other cancer types. For instance, aloperine treatment inhibits proliferation and induces apoptosis of human breast cancer line via blocking Ras/Erk signaling (Tian et al., 2018); as well as for thyroid cancer cell lines, multiple myeloma cell lines and osteoscarcoma MG-63 and U2OS cell lines, inactivation of PI3K/Akt pathway is predominantly involved (Wang et al., 2015; Chen et al., 2018; Lee et al., 2018). Moreover, aloperine administration produced potent effects against HCT116 colon cancer cell line in a dose and time dependent manner, indicating a promising chemotherapeutic potential in human colon cancer (Zhang et al., 2014).

Tumor progression is a multi-factorial process, which involves interaction between tumor cells and immune cells in the intricate tumor microenvironment (Wu and Dai, 2017; Altorki et al., 2019). An alternative strategy for cancer treatment is to unleash immune inhibitory signals and re-energize the cytotoxicity of tumor-killing NK and CD8^+^ T cells. SA-49, a novel aloperine derivative, decreases the expression of PD-L1 on non-small cell lung cancer cells through promoting the translocation of PD-L1 for lysosomal degradation and subsequently enhancing the cytotoxicity of co-cultured T and NK cells. In Lewis tumor xenograft model, the tumor-killing effect of SA-49 was further testified (Zhang et al., 2019). These data unveiled a unique anti-tumor function of aloperine, which is distinct from that of other herbal compounds and conventional immune check-point inhibitors.

### Aloperine Inhibits Tumor Metastasis

Aloperine executes antitumor effect through direct killing and also the inhibition of tumor migration and metastasis. The metastatic process, which is mediated by matrix metalloproteinases (MMPs), plays a vital role in the development of various cancers and is positively linked to the poor prognosis of cancer patients (Wan et al., 2013; Li K. et al., 2020). Thus, interference of tumor metastasis is crucial to tumor therapy, especially for the malignant subtypes. Gao and Tian’s group found that aloperine treatment remarkably suppresses the invasive capability of osteosarcoma and breast cancer cells through down-regulating the expression level of MMP-2/9 (Chen et al., 2018; Tian et al., 2018). Nonetheless, the research on metastasis part is relatively inadequate compared to that of survival field; thus, more future studies are needed to clarify the metastasis-inhibition function of aloperine.

In conclusion, aloperine, when used alone or in combination with other chemotherapeutic drugs, is effective in fighting against various cancers, including hepatoma, colon cancer, prostate cancer, breast cancer, thyroid cancer, osteosarcoma and leukemia. This wide range of anti-tumor effect is mainly accomplished through apoptosis induction, cell cycle arrest, growth inhibition and suppression of migration, which hinder the survivability and metastasis of tumor cells. Although the definite interacting molecules and more detailed mechanisms remain to be determined, undoubtedly, aloperine and its derivatives could act as promising candidates in cancer treatment.

## Anti-Microbial Effect of Aloperine and Its Derivatives

**FIGURE 2:**
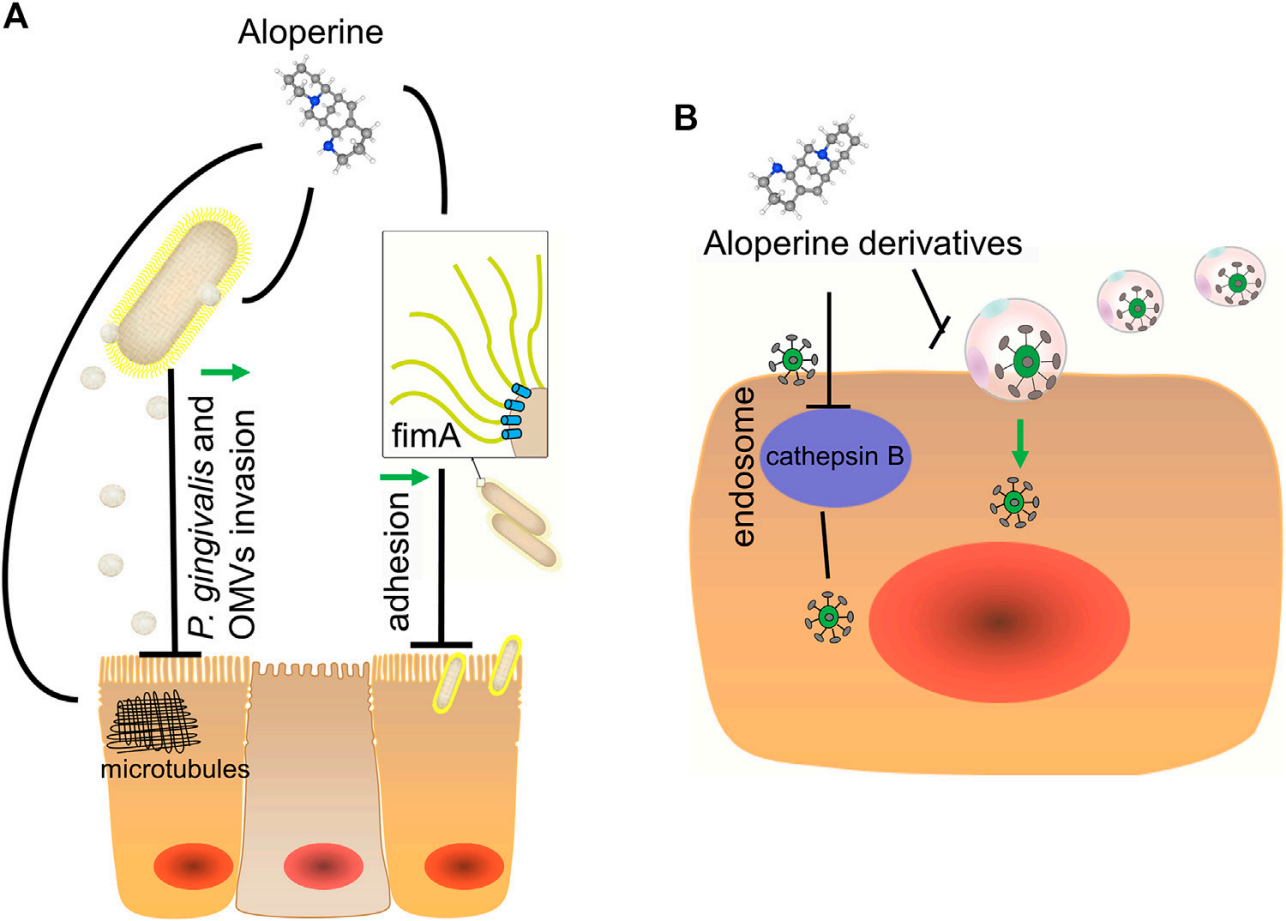
Anti-microbial effect of aloperine. **(A)** Aloperine blocks the entry of *P. gingivalis* and its outer membrane vesicles (OMVs) into oral keratinocytes through inducing unique microtubule arrangement of host cells and inhibiting expression of firmA of *P. gingivalis*. **(B)** Aloperine hinders the stage of viral entry via various mechanisms. On the one hand, aloperine could directly disrupt protein mediated cell-cell fusion. On the other hand, aloperine targets certain proteins in host cells to prevent infection, such as cysteine cathepsin B.

*Sophora alopecuroides L.* has been traditionally used as an antimicrobial agent, the function of which can be largely attributed to the broad-spectrum antibacterial and antiviral activities of aloperine (Shi et al., 2003). As a quinolizidine alkaloid extracted from *Sophora alopecuroides L*., aloperine possesses significant nematocidal and insecticidal activities via binding to the nicotinic acetylcholine receptor (Liu et al., 2008). Ho et al. firstly revealed the potent activity of aloperine against *P. gingivalis* invasion and the effect on its outer membrane vesicles formation (Ho et al., 2016). The mechanisms are related to the regulation of microtube arrangement and expression of fimA, a major form of fimbriae that is necessary for *P. gingivalis*’ attachment to oral surface and co-adhesion with other oral bacteria (Figure 2A).

Peng’s group demonstrated that, through inhibition of endocytosis, aloperine effectively prevents the propagation of hepatitis virus C (HCV) in Huh7.5 cell line and primary human hepatocytes without showing cytotoxicity (Lv X. et al., 2020). Zhang et al. took aloperine as a leading structure to design the compound 7f, a derivative displaying good oral pharmacokinetics and safety profile while exhibiting the potential potency with low EC50 value (micro-molar level). Compound 7f is effective in fighting against both wild type and direct-acting antiviral agents-resistant HCV variants via targeting on viral entry stage (Zhang et al., 2018b). Similarly, Chen’s group identified aloperine as a novel anti-HIV agent. At the same time, they optimized the structure of aloperine to acquire “compound 19” that possesses markedly improved anti-HIV activity. Mechanistic study revealed that “compound 19” prevents the virus from fusing with the host cell membrane rather than inhibiting the binding of HIV-1 to its receptors (Dang et al., 2017). Interestingly, their group also got a series of analogues of aloperine that have improved anti-Influenza A Virus (IAV) activity through targeting nucleoprotein of IAV with a different mechanism of action from that of oseltamivir, a first-line anti-IAV drug (Table 2) (Dang et al., 2014). In addition, Zhang et al. introduced a N^12^ dichlorobenzyl group to aloperine to obtain a new drug named “compound 2e”, which exhibits the most potent anti-ebola virus and anti-marburg virus effect both *in vitro* and *in vivo*. Compound 2e could block the late stage of viral entry, mainly through inhibiting cysteine cathepsin B activity of host components (Figure 2B) (Zhang et al., 2018a).

**TABLE 2:** Improved activities of midified aloperine derivatives.

Modification Position		Name	Improvement	Reference
C16 = C17	N12			
Unsature	—	aloperine	—	(Dang et al., 2014)
Sature	H	Dihydroaloperine	Slightly more potent anti-influenza virus	
Unsature	N-methyl	Compound 9	Improved anti-Influenza A virus (H1N1, H3N2)	
Sature	N-methyl	Compound 17		
Unsature	N-ethyl	Compound 10		
Sature	N-ethyl	Compound 18		
Unsature	(CH2)4-NH- C(=O)CH2-CF_3_SO_2_C_6_H_4_	Compound 19	Enhanced anti-HIV potency	(Dang et al., 2017)
Sature	(CH2)4/5-NH-CO-trifluoromethoxy-benzamide	Compound 6d/6c		
Sature	(CH2)4-NH-CO-trifluoromethyl-benzamide	Compound 12d		
Unsature	3′,4′-Cl_2_PhCH_2_	Compound 2e	Broad-spectrum anti-filovirus profiles against both EBOV and MARV	(Zhang et al., 2018a)
Unsature	12N-4′-methylpiperazine-10-sulfomyl	Compound 7f	Higher pharmacokinetic and safety profile improved anti-HCV activity	(Zhang et al., 2018b)
Unsature	1-methyl-1H-imidazol-4-yl-sulfonyl	SA-49	Decreased the expression of PD-L1 in non-small cell lung cancer cells	(Zhang et al., 2019)

EBOV, Ebola virus; MARV, Marburg virus; HCV, Hepatitis C virus; HIV, Human immunodeficiency virus.

In brief, aloperine not only represents a novel antibacterial and antivirus agent, but also provides a privileged scaffold which can be further optimized by introduction of different chemical modifications.

## Cardiovascular Protection Effect of Aloperine

Cardiovascular disease (CVD) increasingly becomes a public health issue that ranks 1 of the leading causes of death (Roth et al., 2017; Zhao et al., 2019). The prevalence of CVD and its comorbidities poses a huge challenge for modern medicine, thus, there is an urgent need to develop novel effective therapeutic and prophylactic agents (Joseph et al., 2017). Many TCMs, like Danshen, Sanqi and Chuanxiong etc., are commonly used in CVD treatment (Li et al., 2012; Liu and Huang, 2016; Wang et al., 2017). Here, we emphasize the protective effects of aloperine on various CVD complications, which may provide useful hints for future drug screening work.

### Anti-Atherosclerosis Effect of Aloperine

Atherosclerosis (AS) is a common CVD characterized by arterial inflammation and stenosis (Herrington et al., 2016). The pathogenesis of AS involves multiple factors, such as hyperlipidemia, endothelial cells damage, foam cell formation as well as excessive proliferation and migration of vascular smooth muscle cells (VSMCs) (Zimmer et al., 2015). Several studies demonstrated that Compound Kudouzi could lower blood lipid level in high fat diet induced rat AS model (Wang and Chen, 2006; Liu and Chen, 2014; Wang et al., 2020). Moreover, a recent study reported that aloperine administration provides protection from oxidized LDL induced injuries and inhibits the adhesion of U937 monocytes to HUVECs via reducing the expression of IL-6, MCP-1, VCAM-1 and E-selectin (Li Y. et al., 2020). Decreased oxidative stress may also contribute to the beneficial effect of aloperine. However, the related evidence is still insufficient and more *in vivo* studies remain to be carried out to elucidate whether these activities are authentically reliable.

### Anti-Hypertension Effect of Aloperine

Hypertension, frequently accompanied by dyslipidemia and hyperglycemia, is so far the most important trigger of CVD (Van Kleef and Spiering, 2017). Commonly used antihypertensive drugs include ACE inhibitors, alpha blockers, calcium channel blockers, diuretics and vasodilators with distinct modes of action (Laurent, 2017). The hypotensive effect of natural herbs has gained increasing attention due to their unique advantages, such as the multi-target efficacy, safety as well as low cost (Tabassum and Ahmad, 2011).

#### The Vasodilation Effect of Aloperine

Generally, vasodilation agents are the first-line drugs in hypertension treatment. For example, as a calcium channel blocker, amlodipine reduces intracellular calcium concentration and leads to arterial smooth muscle relaxation (Little and Cheng, 1994). However, few antihypertensive drugs that target on potassium ion channels have been reported.

For the first time, Zhou’s group demonstrated that aloperine shows a vasodilation effect in rat thoracic aortic rings via an unknown mechanism (Yang et al., 2018). To make a step forward, Abbott et al. revealed that aloperine is a potassium voltage-gated channel subfamily Q member (KCNQ)-dependent vasorelaxant that isoform selectively activates potassium voltage-gated channel subfamily Q member (KCNQ5) by binding to the “foot” of the potassium channel voltage sensor (Manville et al., 2019). Although several extracts from *Sophora flavescens* confer vasodilatory effect, including aloperine, matrine, and oxymatrine, Abbott suggested that aloperine is the specific KCNQ5 activator through directly binding to KCNQ5 R212 site. These studies not only identified aloperine as a potential agent in hypertension treatment, but also provide examples for the understanding of TCM theory from a perspective of modern medicine.

#### Aloperine Inhibits Vascular Remodeling

Chronic high blood pressure induces vascular and cardiac remodeling, which then push CVD into an irreversible stage. Abnormal proliferation, migration and apoptosis resistance of VSMCs play important roles in vascular remodeling (Harvey et al., 2016; Brown et al., 2018). Besides, under external stimuli, activated NF-κB and NOX activity subsequently accelerate the disease progression (Montezano et al., 2015).

Wu et al. reported that intragastric administration of aloperine exerts protective effect on pulmonary hypertension (PAH) induced by monocrotaline, which was characterized by decreased collagen deposition and improved PAAT and PAD parameters (Wu et al., 2017a). On the one hand, aloperine significantly suppresses the proliferation of pulmonary arterial VSMCs that act as driving force in the initiation and development of PAH (Chang et al., 2019). After treatment, aloperine inhibits NF-κB pathway activation, resulting in an increase of p27^kip1^ and down-regulation of cyclin E1. On the other hand, aloperine decreases the expression of NOX-2 and NOX-4 and subsequently, oxidative stress induced vasoconstriction is abolished. Moreover, the PAH ameliorating effect can also be achieved through aloperine mediated inhibition of RhoA/ROCK signaling, which is crucial for the recruitment of myofibroblast (Wu et al., 2017b). Altogether, these studies support the idea that aloperine is helpful in hypertension treatment.

### Myocardial Protection Effects of Aloperine

**FIGURE 3:**
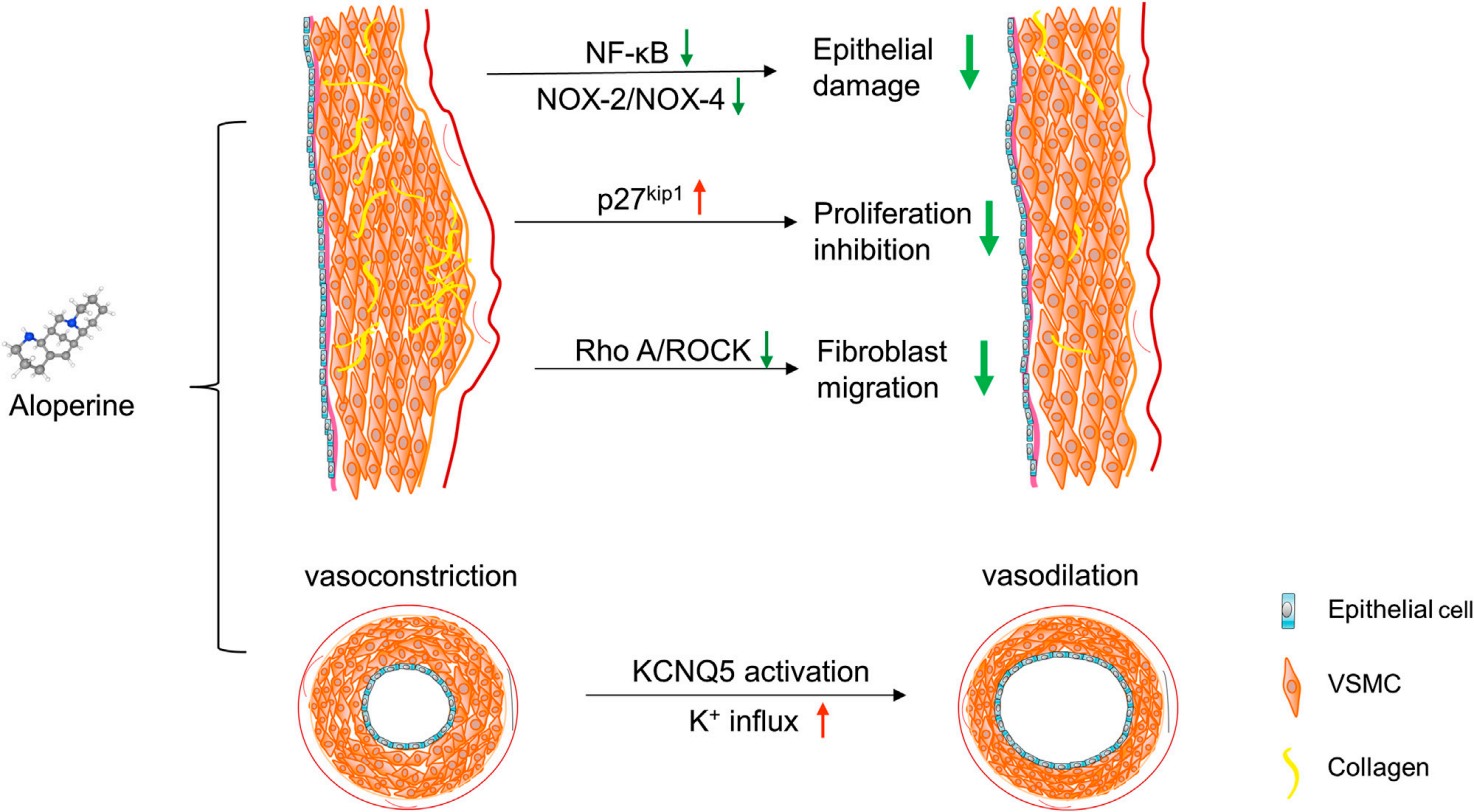
Cardiovascular-protection effects of aloperine. Aloperine exerts regulatory function on different cell types. Aloperine ameliorates endothelial damage by decreasing NOX2/4 and inhibiting NF-κB pathway. Aloperine alleviates hypertension-associated vascular remodeling via inhibiting proliferation and migration of vascular smooth muscle cells (VSMCs) and fibroblasts. In addition, aloperine directly binds with KCNQ5 expressed on VSMCs, leading to K^+^ influx and subsequent vessel dilation.

Accumulating studies have revealed the heart protection effect of aloperine. Mao et al. demonstrated that aloperine administration attenuates cardiac dysfunction induced by coronary micro-embolization, which is indicated by decreased serum cTnI and reduced myocardial infarction area. Such myocardial protection effect was related to the activation of PI3K/Akt signaling pathway and subsequent reduction of myocardial apoptosis (Mao et al., 2019). However, it is contradictory to the conclusion of other studies implying a PI3K/Akt inhibitory role of aloperine, and this discrepancy is possibly due to different cell types. In addition, it is reported that aloperine, at appropriate dose, may be useful in the treatment of cardiac arrhythmia via unknown mechanism (Zhao et al., 1986; Feng and Zhou, 2000; Li Y. et al., 2020). Given KCNQ5 is also expressed on myocardial cells, whether the anti-arrhythmic action is attributed to the activation of potassium channels remains to be determined. Taken together, aloperine exhibits potent cardiovascular protection effect by acting on multiple pathways (Figure 3). As a candidate drug, aloperine is worthy of in-depth researches in the future.

## Anti-Oxidative Stress and Immune Regulatory Effect of Aloperine

### Aloperine Attenuates Oxidative Stress

Oxidative stress impairs many cellular processes and plays critical role in disease conditions such as aging, diabetes and neurological disorders (Dandekar et al., 2015; Cabello-Verrugio et al., 2017). Published work demonstrated that aloperine could confer organ protective effect, which is attributed to its anti-oxidation and anti-inflammation characteristics.

Previous study found that aloperine alleviates neuropathic pain induced by chronic constriction injury, which is related to the reduction of reactive oxygen species (ROS) via suppression of NF-κB pathway (Xu et al., 2014). The up-regulation of TNF-α, IL-6 and IL-1β induced by chronic constriction injury in the dorsal spinal cord was remarkably reversed at the dosage of 80 mg/kg. In addition, Ma’s study showed that aloperine treatment (25, 50, and 100 mg/kg) attenuates neuronal damage induced by oxygen/glucose deprivation and reperfusion, evidenced by increased cell viability and decreased cell morphologic impairment (Ma et al., 2015). Mechanistically, aloperine reduces intracellular malondialdehyde content and bolsters the antioxidant enzymatic activity of catalases, including superoxide dismutase and glutathione peroxidase. Zhao et al. investigated the effect of aloperine on A β-induced neuronal oxidative insults *in vitro*. They found that aloperine treatment ameliorates oxidative stress in N2a/Swe.D9 cells by reducing the production of ROS and 4-HNE, both of which are important biomarkers in the brain of AD patients (Zhao et al., 2018). What’s more, another study showed that aloperine can ameliorate oxidative damage of early brain injury following subarachnoid hemorrhage, most likely through the Nrf2-ARE pathway (Song et al., 2018).

In our study conducted before, we identified that aloperine could protect mice against ischemia reperfusion induced renal injury via regulating mTOR pathway and AP-1 activity (Hu et al., 2016). Aloperine enhances superoxide dismutase expression to promote ROS detoxification and to correct the imbalance between Bcl-2 and Bax. Yin et al. showed that aloperine could significantly mitigate bleomycin-induced pulmonary fibrosis by attenuation of fibroblast proliferation and differentiation through repressing PI3K/AKT/mTOR and TGF-β/Smad signaling, respectively (Yin et al., 2018). Taken together, these results suggest that aloperine may act as an important therapeutic agent in oxidative stress related diseases.

### Immune Regulatory Function of Aloperine

Based on TCM theory, bitter food or medication, such as snake gall and coptis, possess anti-inflammatory function. This is also the case on aloperine, which displays excellent immune modulatory property (Zhou et al., 1989; Lin and Lin, 2011). Wang et al. revealed that aloperine attenuates allergic airway inflammation by lowering inflammatory cell infiltration, down-regulating IL-4, IL-5 and IL-13 expression, and reducing goblet cell hyperplasia (Wang et al., 2018). In addition, aloperine directly abrogates LPS-induced NO and PGE2 production in RAW264.7 cells by suppressing iNOS and COX-2 activity (Ye et al., 2020). Allergic contact dermatitis is a delayed-type hypersensitivity reaction mediated by hapten-specific T cells. Guo’s group demonstrated that topical 1% aloperine cream treatment suppresses 2, 4-dinitrofluorobenzene induced ear swelling, ear erythema as well as the secretion of inflammatory cytokines like TNF-α, IL-1β and IL-6 (Yuan et al., 2010; Yuan et al., 2011).

Regulatory function of natural plant derived small molecules on immune cells, especially T cells, is an interesting research area. Previous study revealed that total alkaloids of *Sophora alopecuroides* could increase CD4^+^CD25^+^Treg cell proportion and IL-10 level in rats, indicating a potential immunomodulatory capacity of aloperine (Zhou et al., 2010). Next, it is verified that aloperine promotes the expression of key Treg transcription factor Foxp3 via suppressing PI3K/Akt/mTOR signaling and glycolysis pathway in dextran sodium sulfate elicited colitis model (Fu et al., 2017). However, additional possible regulatory roles of aloperine on T cell biology still requires further study.

In conclusion, these studies proved that aloperine possesses excellent anti-oxidation and anti-inflammation properties, indicating its potential use for prevention or treatment of various associated disorders in clinic.

## Discussion

**FIGURE 4:**
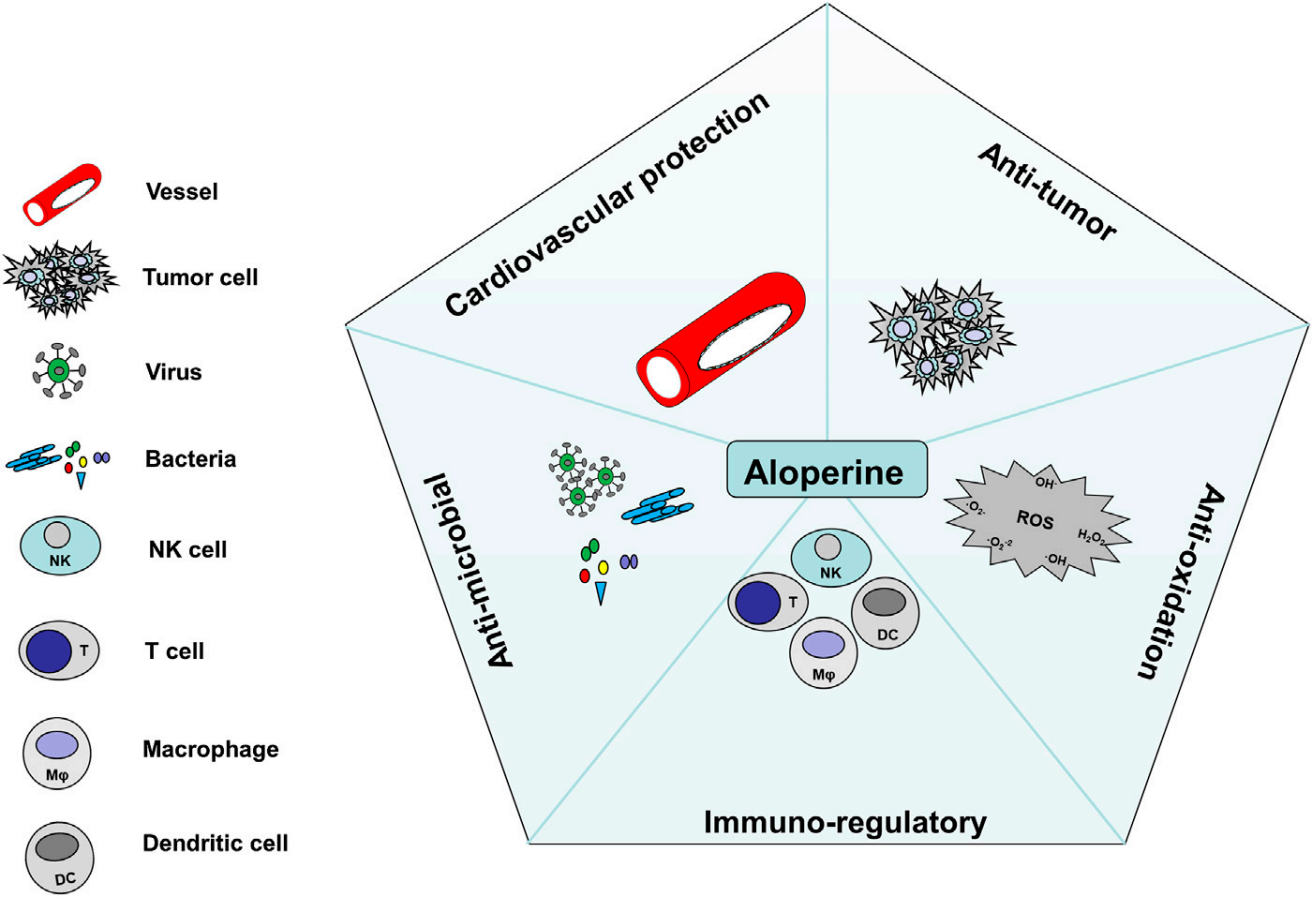
Schematic representation of pharmacological activities of aloperine.

During past decades, modern medical technology has brought unprecedented advance to folk medicine research. Importantly, it is a good method to screen appropriate and effective drugs through combination of the theory of TCM and modern understanding of different diseases, and the shining example is artemisinin. Interest in the alkaloids stems from the wide variety of physiological effects it produces in humans and other organisms. In the present review, we concluded the pharmacological effects and associated mechanisms of aloperine, which make it a potential candidate for the treatment of cancer, infectious diseases, cardiovascular complications and inflammation-related disorders (Figure 4).

Like many other alkaloids, evidence suggests that aloperine exerts therapeutic effect on various cancer types such as colon cancer, prostate cancer, thyroid cancer and breast cancer etc. Mechanistically, aloperine induces cell apoptosis and suppresses tumor migration through various signaling pathways including Ras/Erk, PI3K/Akt and MMPs. Intriguingly, Zhang et al. found that, SA-49, a novel sulfonyl-substituted aloperine analogue, down-regulated the protein level of PD-L1 in non-small cell lung cancer cells by promoting melanogenesis associated transcription factor mediated lysosomal proteolysis via PKCα-GSK3β signal pathway. Besides, SA-49 enhances the tumor cell killing capacity of T and NK cells, as determined in C57BL/6 mice bearing Lewis tumor xenografts. Altogether, aloperine serves as an effective anti-tumor agent which has multifaceted roles in cancer treatment. As for CVD, aloperine administration ameliorates cardiac dysfunction caused by coronary micro-embolization partially through the inhibition of myocardial apoptosis. Further study revealed that aloperine suppresses isoprenaline induced cardiac hypertrophy and the mechanism is related to its antioxidant property (Cao et al., 2000). In addition, by systematic network pharmacology analysis, Zhu’s group unveiled that the anti-CVD effect of aloperine may also be related to the modulation of nitrogen metabolism (Huang et al., 2020). Good blood pressure management is essential to reduce morbidity and mortality in CVD patients. Conventional antihypertensive drugs including ACE inhibitors, alpha blockers, diuretics, calcium channel blockers, angiotensin II receptor antagonists and vasodilators are developed with distinct physiological mechanisms and accompanying side effects, respectively. Therefore, it is worthy to note that a great many folk medicines have been used to treat hypertension while the underlying mechanisms are not clear. Recently, Abbott identified aloperine as an activator of KCNQ5 potassium channel. Aloperine could bind to KCNQ5 R212 site and exert vasodilation effect by reducing cell membrane depolarization. Moreover, other tested materials extracted from hypotensive plants such as *Lavandula angustifolia*, *Matricaria chamomilla*, and *Thymus vulgaris* can work in a similar manner. This discovery paves a new avenue for the identification of potential antihypertensive drugs, and perhaps, more specific and efficient aloperine-derived small molecules would be developed with ideal clinical implications.

The core structure of aloperine is the quinolizidine ring (Figure 1B) belonging to a rare family of C^15^ lupine alkaloid. For this reason, aloperine provides a skeletal structure that can be easily modified for further optimization. Danie li’s group developed a practical protocol for synthesis of aloperine in 12 steps starting from the commercially available piperidine-2-ethanol (Passarella et al., 2002). It is reported that the aloperine derivative with either an N-(1-butyl) 4-trifluoromethoxy benzamide side chain or the introduction of trifluoromethane sulfonyl group shows more potent anti-HIV activity. Besides, addition of a sulfonyl moiety on the N^12^ atom results in an enhanced potency against both wild type and direct-acting antiviral agents-resistant HCV variants with more satisfying pharmacokinetic and safety profile. SA-49, a derivative of aloperine, could induce melanogenesis associated transcription factor dependent lysosomal degradation of PD-L1, thus disrupting immune tolerant tumor microenvironment. The reported modifications of aloperine and its improved activities are summarized in detail (Table 2). From the perspective of recognition to practice, modification of aloperine not only gives the drug a chance for better performance, but also provides a deeper understanding of the structure-function interrelationship.

In spite of these excellent properties, clinical application of aloperine has been limited by some inherent shortcomings. One issue to concern falls into the toxicity of aloperine. A recent study by Qiu M et al. pointed out that intraperitoneal (i.p.) aloperine administration induces damage in liver and kidney at the dosage of 16 mg/kg, manifested by cytoplasm vacuolization and swelling respectively (Qiu et al., 2020a). However, after withdrawing aloperine, mice would partially recover at 1 week and fully recover at 4 weeks, which indicates that the kidney and liver injury induced by high dose i.p injection of aloperine is reversible. On our hand, we found that no cytotoxicity was observed until the dosage reaches 150 mg/kg by oral gavage for consecutive 7 days (Hu et al., 2016). Moreover, several studies showed that injection of total alkaloids of *Sophora alopecuroides* is effective in treating diseases including psoriasis and internal hemorrhoid without obvious side effect. Nevertheless, the pharmacokinetic properties of aloperine remain to be determined *in vivo*. Another drawback comes to its poor solubility in aqueous solvents. The increased dosage attributed to compromised bioavailability would exaggerate the toxicity to body system. Thus, strategies for improving aloperine water-solubility are demanded. Theoretically, the modification of parent structure of aloperine would directly improve the hydrophilicity, for example, the introduction of a hydrophile component. However, till now, the related studies are scarce and no feasible solution is proposed. Specific drug delivery systems may be an alternative option to overcome these obstacles and to increase the efficacy at the target location (Liu and Feng, 2015; Xu et al., 2015; Vader et al., 2016). Among them, nano-capsule based technology gains increasing attention. Many materials such as polyethylene glycol, polysaccharide, chitosan and liposome were used as nano-capsules to efficiently encapsulate the purified bioactive cargo for drug delivery through intravenous injection or oral administration (Kolate et al., 2014; Li et al., 2019). Altogether, tremendous efforts are needed to improve the solubility and selectivity of aloperine for future clinical application.

In conclusion, aloperine holds the potential to be developed into a novel multifunctional drug due to its various bioactivities and safety. However, more scientific evidence based on clinical and animal studies is eagerly needed.

## Author Contributions

HZ, JL, and FS proposed and wrote the manuscript. FW, ML, and YD collected and analyzed the information. DH and HF supervised the conception and writing of the article.

## Funding

The research was supported by the National Natural Science Foundation of China (Grant No. 31770983, 81974249).

## Conflict of Interest

The authors declare that the research was conducted in the absence of any commercial or financial relationships that could be construed as a potential conflict of interest.
